# Leveraging community health worker system to map a mountainous rural district in low resource setting: a low-cost approach to expand use of geographic information systems for public health

**DOI:** 10.1186/1476-072X-13-49

**Published:** 2014-12-06

**Authors:** Fabien Munyaneza, Lisa R Hirschhorn, Cheryl L Amoroso, Laetitia Nyirazinyoye, Ermyas Birru, Jean Claude Mugunga, Rachel M Murekatete, Joseph Ntaganira

**Affiliations:** College of Medicine and Health Sciences, University of Rwanda, Kigali, Rwanda; Partners In Health/Inshuti Mu Buzima, Kigali, Rwanda; Partners In Health, Boston, MA USA; Department of Global Health and Social Medicine, Harvard Medical School, Cambridge, MA USA; Ariadne Labs, Boston, MA USA; Center of Geographic Information System and Remote Sensing, University of Rwanda, Kigali, Rwanda; College of Science and Technology, University of Rwanda, Kigali, Rwanda

**Keywords:** GPS, Community health workers, GIS, Costing, Disparities, Resource limited settings, Drinking water sources

## Abstract

**Background:**

Geographic Information Systems (GIS) have become an important tool in monitoring and improving health services, particularly at local levels. However, GIS data are often unavailable in rural settings and village-level mapping is resource-intensive. This study describes the use of community health workers’ (CHW) supervisors to map villages in a mountainous rural district of Northern Rwanda and subsequent use of these data to map village-level variability in safe water availability.

**Methods:**

We developed a low literacy and skills-focused training in the local language (Kinyarwanda) to train 86 CHW Supervisors and 25 nurses in charge of community health at the health center (HC) and health post (HP) levels to collect the geographic coordinates of the villages using Global Positioning Systems (GPS). Data were validated through meetings with key stakeholders at the sub-district and district levels and joined using ArcMap 10 Geo-processing tools. Costs were calculated using program budgets and activities’ records, and compared with the estimated costs of mapping using a separate, trained GIS team. To demonstrate the usefulness of this work, we mapped drinking water sources (DWS) from data collected by CHW supervisors from the chief of the village. DWSs were categorized as safe versus unsafe using World Health Organization definitions.

**Result:**

Following training, each CHW Supervisor spent five days collecting data on the villages in their coverage area. Over 12 months, the CHW supervisors mapped the district’s 573 villages using 12 shared GPS devices. Sector maps were produced and distributed to local officials. The cost of mapping using CHW supervisors was $29,692, about two times less than the estimated cost of mapping using a trained and dedicated GIS team ($60,112). The availability of local mapping was able to rapidly identify village-level disparities in DWS, with lower access in populations living near to lakes and wetlands (p < .001).

**Conclusion:**

Existing national CHW system can be leveraged to inexpensively and rapidly map villages even in mountainous rural areas. These data are important to provide managers and decision makers with local-level GIS data to rapidly identify variability in health and other related services to better target and evaluate interventions.

## Background

The spread of Geographic Information Systems (GIS) – a set of tools to capture, store, transform, analyse, and display spatial data –has improved spatial analysis of health related services and population health [1–4]. Health data combined with geographic information allows us to analyse the spatial variation of diseases burden, mortality, morbidity, physical access to health care and social or environmental determinants of health outcomes [1, 2, 5–9]. The transformation of detailed data into maps can facilitate communication of geographic distribution of health challenges in different communities and identify areas for intervention [4, 5, 10].

The use of GIS in low resource settings has been hampered by a number of factors including data availability, software, expertise, and economic resources needed [4, 6, 11]. Participatory mapping is a map production process by local communities with the support of governmental institution, non-governmental organizations (NGOs) or academic institutions [12]. The United Nations (UN) Millennium Development Goals (MDGs) and the World Summit on the Information Society suggested utilization of a participatory approach to promote equal access to information and knowledge sharing [13]. This approach could offer a relatively low resource method to map communities in rural settings.

Partners In Health/Inshuti Mu Buzima (PIH/IMB) has partnered with the Rwandan Ministry of Health (MoH) since 2005 to provide support to three districts (Kayonza, Kirehe and Burera) in rural Rwanda, including supporting the community health workers (CHW) system to provide community-based care. Since 2009, PIH/IMB has used GIS to supplement existing monitoring and evaluation efforts in supported areas to better target district-wide health system strengthening interventions [14]. Village location mapping was done using a trained GIS team of PIH/IMB in Kayonza (Rwinkwavu District Hospital catchment area only) and Kirehe districts and was relatively resource intensive. In the third district, Burera, a mountainous rural district with poor road access, we adapted a community participatory approach to conduct village-level mapping. We describe this approach of leveraging Rwanda’s national CHW program to map village locations, and demonstrate the utility of this process through identification of geographic disparities in critical services through mapping of village-level access to safe water.

## Methods

**Figure 1:**
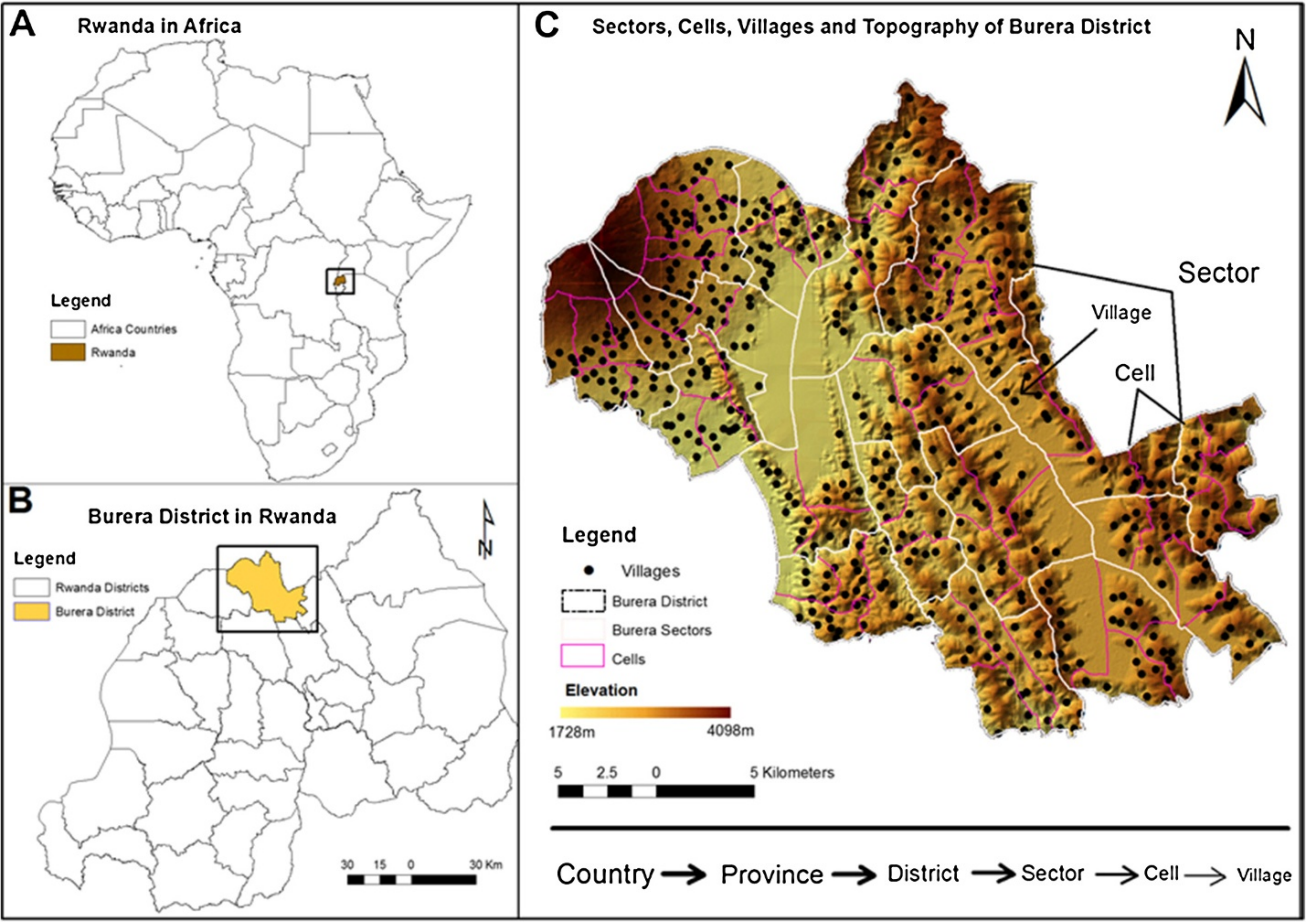
**Location of the study area as well as its topography, sectors, cells, and villages. A**: location of Rwanda in Africa, **B**: location of Burera district as one of 30 district of Rwanda, **C**: Burera district subdivisions; 17 sectors, 69 cells and 573 villages, and the Digital Elevation Modal (DEM) showing elevation and terrain of Burera district.

Study area: Burera district, one of the 30 districts of Rwanda, is located in the Northern Province, neighboring Uganda. Burera is a mountainous rural district with a topography ranging between 1728 m to 4098 m of altitude above sea level with an area of 646 km^2^ (Figure 1). The district is subdivided by Rwandan administrative boundaries into 17 sectors, with 69 sub-sector divisions (cells) and 573 villages, with each cell containing between five and 16 villages (Figure 1).

Rwanda National CHW network: Each village has four CHWs in charge of community health and each cell has a CHW supervisor. CHW supervisors live in the community, and have completed a minimum of primary school education. Each health center (HC) or health post (HP) is staffed with a nurse who supervises the community health activities in the catchment area.

Community mapping: Four meetings were held for authorities from the district, district hospital (DH), HCs and some CHW supervisors in order to encourage participation and ownership in the mapping process. During these meetings we reviewed the approach and goals of GIS mapping and potential to help efforts to improve health care in Burera district.

Training process: We developed a three day training program and training manual in English and Kinyarwanda to build knowledge around the use of Global Positioning System (GPS) device and the skills needed to collect the data. The first day of the training included the introduction and explanation of the purpose of the activity, the value of map analysis, and an introduction to GPS functionalities. The second day focused on the use of the GPS device; emphasizing taking GPS coordinates points. The last day was field-based practice, where trainees collected GPS coordinates that were then validated, and also discussed challenges and solutions. CHW supervisors who had more difficulty using GPS were identified by trainers and given coaching on their first day of data collection to ensure data quality. In total 111 people were trained, 69 cell-level CHW supervisors, 17 sector-level CHW supervisors, and 25 nurses in charge of community health at HCs and HPs. A full-time Burera-based District GIS project assistant was hired by PIH/IMB who provided to training, validation and support for the CHW supervisors field work and data entry.

CHW staff: All CHW supervisors and nurses in charge of community health at HC and HP level were included in the training. Different roles were attributed to each cadre: cell-level CHW supervisors collected the village GPS coordinates; sector-level CHW supervisors and community health nurses provided supportive supervision and organization of the field data collection along with the GIS project assistant.

GPS data collection: Cell-level CHW supervisors collected GPS coordinates of all villages in their cell over five days. The GPS device was used to collect longitude and latitude information of a specific location, and the collected data was stored as point features in the device. Every data collector had a GPS with two extra fully charged batteries. We started data collection in the central part of the district, and then continued north to the mountainous part of the district in the dry season (July, September, and October) due to transportation-related challenges presented by the rainy season. Village location was mapped based on where population gathered for meeting places like village office or village chief’s house

Drinking Water Sources (DWS) and population data: Information on DWS and population was provided by the elected chief of the village in 2013 through a brief survey administered by the CHW supervisor. DWSs were classified as safe water (water from Improved Drinking Water Source (IDWS)) and unsafe water (water from Unimproved Drinking Water Source (UDWS)) using the World Health Organization (WHO) definitions [15]. IDWSs were defined as adequately protected from outside contaminations, including piped household water connection, public standpipe borehole, protected dug well, protected spring and rainwater collection. UDWSs were those inadequately protected from outside contamination, including unprotected dug well and spring, surface water, vendor-provided water (cart with small tank/drum, tanker truck), and bottled water (bottled water is considered improved only when the household use another improved source for cooking and personal hygiene) [15]. Since there may be multiple drinking water sources in a village, we classified the most frequently used drinking water source as primary and others were classified as secondary.

**Figure 2:**
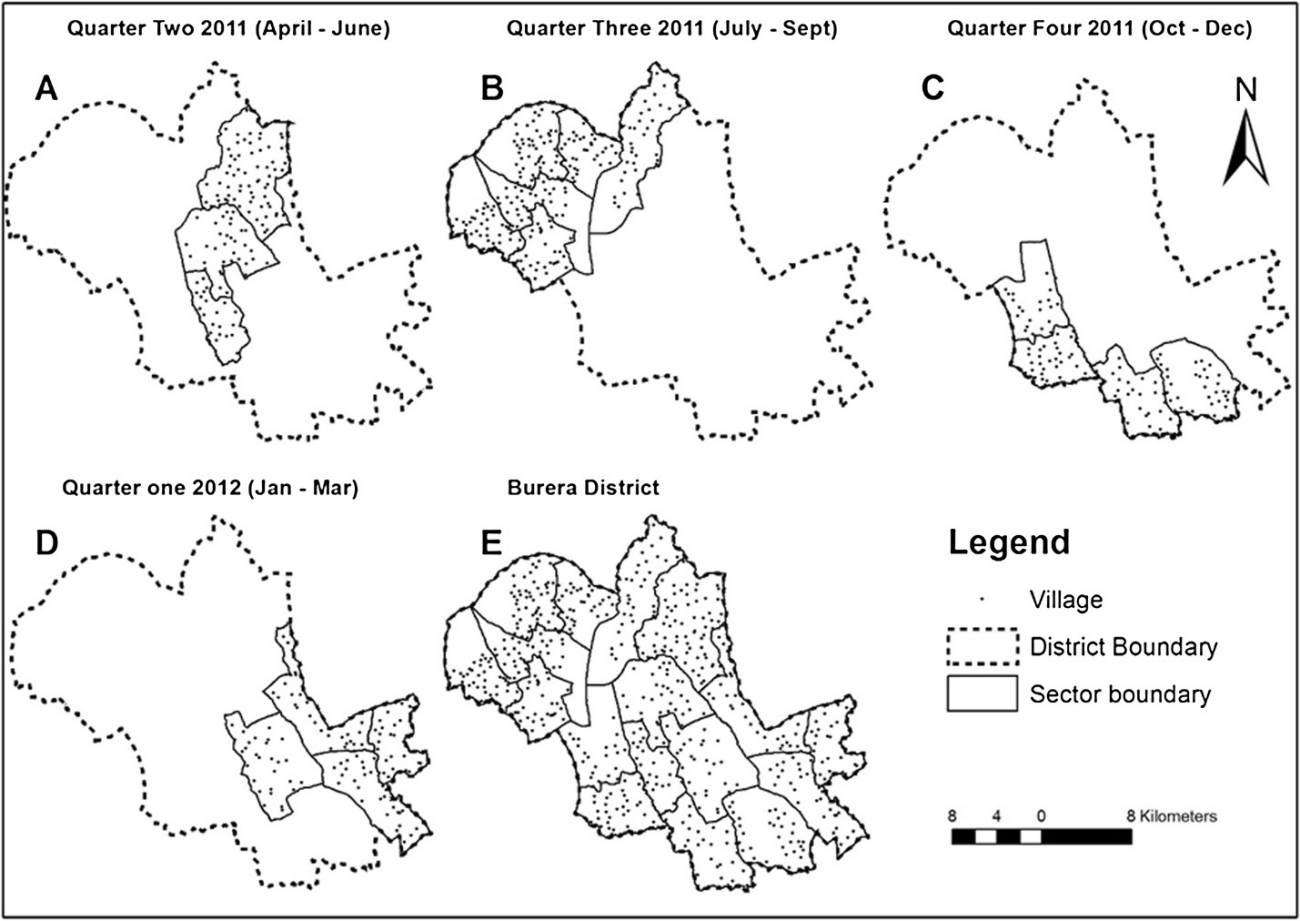
**Villages mapped by quarter. A**: 125 villages (three sectors) in quarter two of 2011 (April – June). **B**: 220 villages (six sectors) in quarter three of 2011 (July – September). **C**: 116 villages (four sectors) in quarter four 2011 (October – December). **D**: 112 villages (four sectors) in quarter one of 2012 (January - March). **E**: Total of 573 villages of 17 sectors in four quarters.

**Figure 3:**
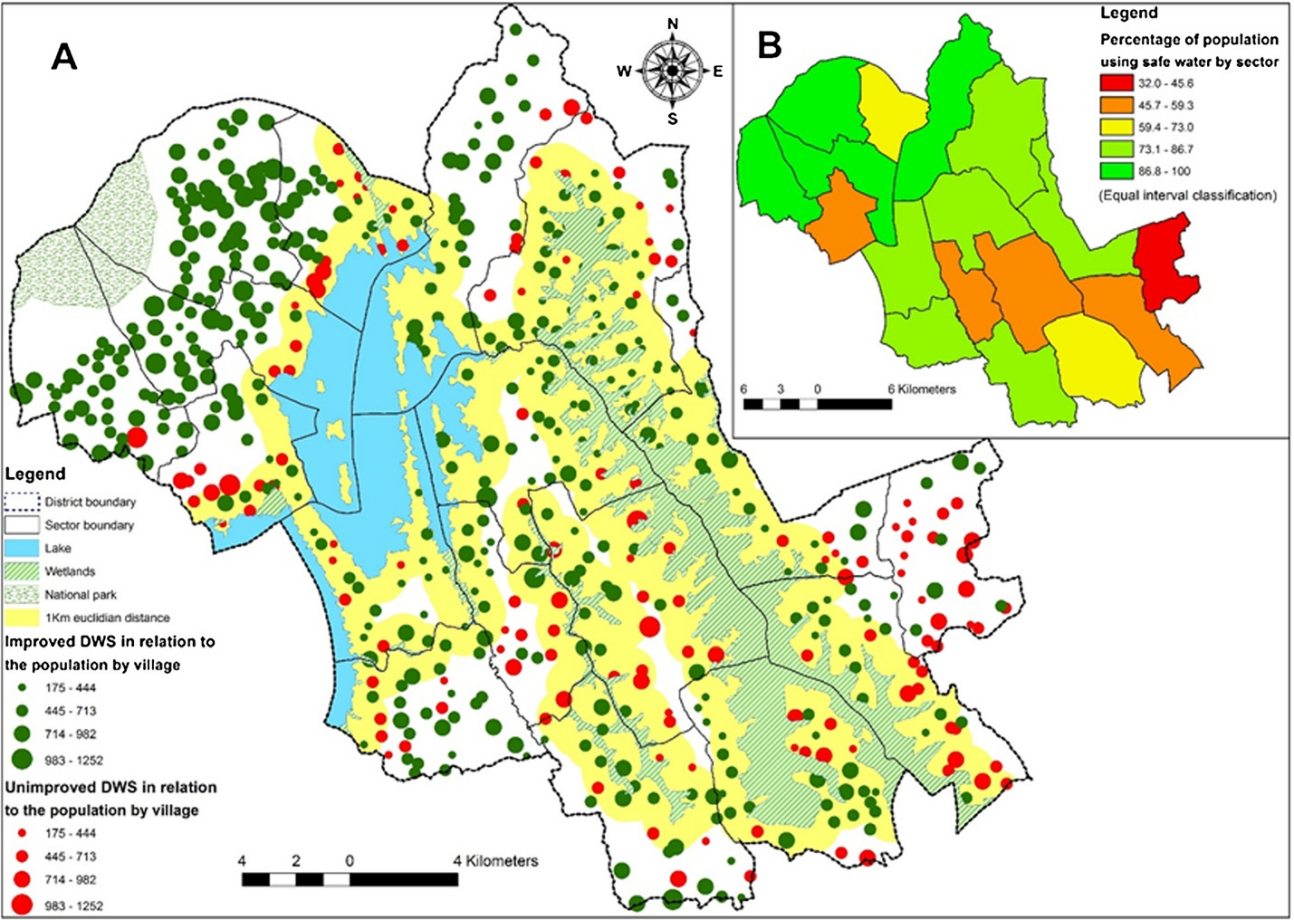
**Drinking water distributions by village and sector. A**: The dot represented the location of villages while the size of dot was proportion to the number of population in the village. The dots in green color represented safe water while dots in red color represented unsafe water. Area in yellow was a one kilometer Euclidian distance from lake in blue and wetland in green and while lines. **B**: Represented the percentage of population using safe drinking water by sector which decreased from green to yellow and red, from 100% (the highest) to 32% (the lowest).

Other data: The list of villages’ names and their corresponding cells and sectors of 2009 was obtained from the Ministry of Local Government (MINALOC). Rwandan country, district, sector boundary, road network shapefiles released in 2006 and lakes, wetlands released in 1992 were collected from University of Rwanda Center of Geographic Information System and Remote Sensing (UR-CGIS). Cell boundary shapefile released in 2008 was obtained from National Institute of Statistics of Rwanda (NISR).Village GPS coordinates were imported as a shapefile using DNR Garmin software (version 5.03.0002). Coordinates were joined to the village’s name, to allow the visualization in the map. Maps were produced (Figures 1, 2 and 3) using geo-processing tool in ArcGIS 10.1. To map village DWS, the shapefile was joined with information on the DWS and population data and combined with district lakes, and wetlands.

Map validation and distribution: CHW supervisor-collected GPS coordinates were first validated by the GIS assistant. He randomly recollected at least two villages GPS coordinates for each cell, and compared them to those collected by the CHW supervisor. Secondly, we met with local authorities from cell, sector, district, HC and DH to validate villages GPS locations mapped. Seventeen sector-level validation meetings were held with 124 authorities’ participants. The district map was then validated by district-level authorities during a validation meeting, including 27 participants (vice mayors and district office authorities in charge of health, land and environment, district hospital administrators and other district officials). Once validated, the maps were printed out, laminated and distributed to the district, sector, cell and health facilities for posting and administrative use.

Cost: Costs of the mapping process were estimated from a health system perspective, using the data from program budgets and financial activity records. Before starting the mapping in Burera district, the GIS team at PIH/IMB had just concluded a similar mapping exercise in Kirehe district using a trained full-time PIH/IMB GIS team. We compared the costs of mapping by the CHW supervisors in Burera, with modelled estimates that would have been incurred if staffing resources similar to those of the Kirehe mapping were utilized. To get the cost of personnel and equipment (transport, computers and other devices), we first estimated their capacity rates in hours available for mapping activities during the entire mapping period (weekday hours minus holidays, time-offs and weekends). The concept of useful-life was used to estimate the depreciation and present value of equipment such as vehicles, computers, and GPSs that last for more than a year. Indirect and overhead costs such as PIH/IMB’s organizational and administrative spending related to this mapping were very minimal in both mapping methods, and were therefore ignored. The main cost categories were training, data collection and mapping, validation, and dissemination (Table 1). Unit and total costs were calculated in Rwandan Francs (RWF) and converted into USD using the median exchange rates of April 2011 to March 2012, actual spending period.

**Table 1:** **Cost of the intervention using CHW supervisors compared to GIS team of PIH/IMB**

Cost category	Items counted in costing	Mapping by CHW supervisors		Mapping by GIS team of PIH/IMB	
		USD	%	USD	%
Trainings and orientation	Training manual, room, training materials, meals, refreshment, transport and certificates	4,335	15%	72	0%
Data collection and mapping	Salary GIS coordinator and assistant, hiring GIS assistant, salary CHW supervisors/5 days, transport (vehicle, fuel, and driver), motorcycle rental, meals, communication, accommodation, software (one year license of Arc GIS), laptops, rain coats, GPS devices*	20,166	68%	54,988	91%
Validation (sector & district levels)	Transport, per diem, meals, refreshment, printing	3,345	11%	3,397	6%
Dissemination	Printing, lamination, transport and per diem	1,846	6%	1,655	3%
**Total**		**29,692**	**100**%	**60,112**	**100**%

*GPS devices which were shared by the mappers.

Mapping village level DWS: DWS was overlaid onto the maps to create a village-level district-wide map of access to safe water. We categorized villages as proximal to lakes and wetlands if they were located within one kilometer Euclidean distance and then used chi squared test for association between lake and wetland proximity and DWS type.

### Ethical consideration

This project was reviewed by the institutional review board of University of Rwanda, College of Medicine and Health Sciences, School of Public Health, and was given exempt status.

## Results

### Mapping and validation

Between April 2011 and March 2012, CHW supervisors successfully mapped the 573 villages in Burera district (Figure 2). During the 18 validation meetings, we received 73 comments (64 comments in sector validation meetings and nine in the district validation meeting). Based on the feedback, locations of 32 villages were recollected and corrected. Following validation, we produced the 17 sector maps and the overall district map, with sector-level maps distributed in hard and soft copy to the 69 cells-level offices, 17 sector-level offices and the 18 HCs and 7 HPs. The overall district map was distributed to the district and DH offices.

### Costs

The total cost of mapping using CHW supervisors was $29,692, compared to the $60,112 cost which would have been needed for mapping using the existing GIS team of PIH/IMB. For the participatory mapping method, data collection and mapping accounted for most of the costs (68%), followed by the training (15%), map validation (11%) and dissemination (6%) (Table 1).

### Use of CHW-system derived maps for rapid assessment of DWS

Three-quarters of Burera’s population had two DWS, with 75.1% of primary DWS categorized as safe (IDWS), and 44.6% for secondary DWS. We found that 94.1% of the population had access to safe water for at least one DWS, with 76.2% having access to IDWS through their primary water source. Over two-thirds (69.1%) of the population had their primary DWS located inside their village.Overlay of the DWS information onto the maps allowed rapid assessment of geographic differences in access to IDWS. For example, access to safe water at the sector level ranged from 32.0% to 100%, with the northwest and central part of the district that had better access to safe water than the southeast part (Figure 3). GIS data also allowed analysis of geographic factors associated with better or worse access to IDWS. For example, villages near to lakes and wetlands were more likely to identify an unsafe source as a primary DWS (29.4% versus 19.5% respectively, p < .001).

## Discussion

We successfully leveraged an existing national CHW network to map a mountainous district in Rwanda at lower cost and with effective engagement of the community and other key stakeholders. Following a three-day practical training and supported by a district-based staff member to provide support, CHW supervisors mapped this mountainous rural district in less than one year despite poor roads and no vehicle access to parts during the rainy season.

There were several advantages to using CHW supervisors to collect the geographic data. They were already travelling by foot to the villages as part of their CHW supervisory activities, so no additional transport was needed and they already knew the physical location of the villages. The approach overcame the transportation challenges facing mapping in rural areas with limited road infrastructure. The participation of CHW supervisors in mapping villages and safe water access was also highly valued by government authorities as part of local capacity building and strengthening integration and participation of the local community in decision making.

Not surprisingly, using CHW supervisors resulted in lower cost compared to using a trained and dedicated GIS team. Using program costing data, we estimated that using PIH/IMB’s GIS team would have been about two times more costly compared to the mapping using CHW supervisors (saving about $30,420). The main difference in cost using the CHW supervisor network approach was in the data collection and mapping largely due to salary and transportation, ($20,166 versus $54,988) (Table 1). The process could have been accelerated if the CHW supervisors had more time to do the mapping, but we worked to integrate into their usual activities so that no additional salaries were needed, increasing the feasibility for replication by groups with limited funds.

Community engagement was another important benefit of our approach. Involvement of local community and authority for decision making is recognized as important in addressing gaps in population health and service [16]. Integrating the community into mapping work has also been successfully used in others settings such as the participatory mapping in Tanzania for malaria project [17]. We found similar engagement in both the planning and validation processes increased the acceptability and value of the maps by district and sub-district authorities.

Village level maps were the first ever produced in the district and were given to local authorities to use them for their daily activities and decision making. The work also resulted in a database that can be used for future geographic analysis at this local level. The assessment of the spatial distribution of safe water by the villages was evidence of this value. For example, we were able to rapidly map and illustrate that safe water was unevenly distributed across villages of the district. The access to safe water that we found (76.2%) was similar to the NISR findings in 2011 (76.7%) for the northern province where Burera district was located [18], providing evidence that the approach was able to provide results consistent with other data sources. These maps also had value to help to inform decision makers. When the maps showing village and sector-level variability in IDWS access were shared with the district authorities, they were able to intervene for sectors with low access to safe water by increasing supply of safe water through the WASH (Water, Sanitation and Hygiene) project on-going in the Burera district of Rwanda [19, 20].

We learned a number of lessons which can inform similar approaches to feasibly integrate GIS into monitoring, evaluation and program planning at the local level. Leveraging existing community-level work has the advantage of both reducing cost as well as increasing the local ownership of the data. In addition, ensuring a rapid feedback loop of resulting maps is important to ensure engagement of local authorities and the community in decision-making. Focusing on building capacity and local ownership throughout the mapping process, supported by practical training and skills-based supervision resulted in engaged CHW supervisors motivated to learn new skills and perform the mapping and participate in data-driven decision making without additional reimbursement. For this new mapping activity, we also found that a locally-based GIS assistant was important to supervise initial data collection and address challenges during the process. While we used a proprietary software, the costs could also be lowered through use of free open source software and open e-learning for geo-analysis tools [21, 22].

There were some limitations to our approach. There were not readily recognized village centers, as village location was mapped based on where population gathered for meeting places like village office or village chief’s house. We did not test the accuracy of maps produced using CHW supervisors versus maps produced using GIS team of PIH/IMB. It was also more difficult to validate the location of villages far from known features such as roads, rivers, lakes, and wetland. In addition, the safe water assessment by village was based on the assumption that the entire village’s population used the primary DWS available within or outside the village. This may have resulted in under or over estimates of access to safe drinking water. Modeling the cost of transport from Kirehe to Burera mapping using PIH/IMB’s GIS team, assumed similar driving conditions like road, topography, etc. which might not be the same case.

## Conclusion

The existing CHW system can be leveraged to inexpensively map rural areas. Involvement of local authorities from health and political sectors ensures community buy-in and ownership of the results for future decision-making. The creation of a village-level GIS database is an important to help local officials identify geographic disparities in access to important resources such as safe water, communicate these challenges through maps, and better target interventions to improve population health and reduce inequity. Our approach of leveraging an existing community health network supported through skills-based training and supervision and focused on local engagement and ownership could be replicated in other resource limited countries with community health worker programs similar to the Rwandan model [23].

## References

[1] Schuurman N, Bérubé M, Crooks VA (2010). Measuring potential spatial access to primary health care physicians using a modified gravity model. Can Geographer.

[2] Chambers R (2006). Participatory mapping and geographic information systems: whose map? Who is empowered and who disempowered? Who gains and who loses?. EJISDC.

[3] McLafferty SL (2003). GIS and health care. Annu Rev Public Health.

[4] Tanser FC, Le Sueur D (2002). The application of geographical information systems to important public health problems in Africa. Int J Health Geogr.

[5] Kaminska IA, Oldak A, Turski WA (2004). Geographical Information System (GIS) as a tool for monitoring and analysing pesticide pollution and its impact on public health. AAEM.

[6] Simarro PP, Cecchi G, Paone M, Franco JR, Diarra A, Ruiz JA, Fèvre EM, Courtin F, Mattioli RC, Jannin JG (2010). The Atlas of human African trypanosomiasis: a contribution to global mapping of neglected tropical diseases. Int J Health Geogr.

[7] Ricketts TC (2003). Geographic information systems and public health. Annu Rev Public Health.

[8] Luo W (2004). Using a GIS-based floating catchment method to assess areas with shortage of physicians. Health Place.

[9] Wen TH, Lin NH, Lin CH, King CC, Su MD (2006). Spatial mapping of temporal risk characteristics to improve environmental health risk identification: a case study of a dengue epidemic in Taiwan. Sci Total Environ.

[10] Chung K, Yang DH, Bell R (2004). Health and GIS: toward spatial statistical analyses. J Med Syst.

[11] Fleming G, Van Der Merwe M, McFerren G (2007). Fuzzy expert systems and GIS for cholera health risk prediction in southern Africa. Environ Model Softw.

[12] Mwanundu S, Fara K (2009). Good Practices in Participatory Mapping.

[13] Vajjhala SP (2005). Integrating GIS and Participatory Mapping in Community.

[14] Sudhof L, Amoroso C, Barebwanuwe P, Munyaneza F, Karamaga A, Zambotti G, Drobac P, Hirschhorn LR (2013). Local use of geographic information systems to improve data utilisation and health services: mapping caesarean section coverage in rural Rwanda. Tropical Med Int Health.

[15] World Health Organization, UNICEF (2012). Progress on Drinking Water and Sanitation: 2012 Update.

[16] Dunn CE (2007). Participatory GIS - a people’s GIS?. Prog Hum Geogr.

[17] Dongus S, Nyika D, Kannady K, Mtasiwa D, Mshinda H, Fillinger U, Drescher AW, Tanner M, Castro MC, Killeen GF (2007). Participatory mapping of target areas to enable operational larval source management to suppress malaria vector mosquitoes in Dar es Salaam. Tanzan Int J Health Geogr.

[18] National Institute of Statistics of Rwanda (2011). EICV3 Thematic Report: Environment & Natural Resources.

[19] Nyirishema R, Mukasine B (2011). Multi-Stakeholder Platform for Sustainable WASH Services: Lessons from Rwanda.

[20] Rwanda SNV (2012). Celebrating Sustainable, Affordable Water and Sanitation Services, Wash Project in Rubavu, Nyabihu, Burera and Musanze.

[21] **Quantum GIS** [http://www.qgis.org]

[22] **E-learning** [http://www.geoforall.org]

[23] Republic of Rwanda (2008). National Community Health Policy.

